# Canine atlantoaxial optimal safe implantation corridors – description and validation of a novel 3D presurgical planning method using OsiriX™

**DOI:** 10.1186/s12917-016-0824-3

**Published:** 2016-09-06

**Authors:** Guillaume Leblond, Luis Gaitero, Noel M. Moens, Alex zur Linden, Fiona M. K. James, Gabrielle Monteith, John Runciman

**Affiliations:** 1 Department of Clinical Studies, Ontario Veterinary College, University of Guelph, Guelph, ON N1G 2W1 Canada; 2 School of Engineering, University of Guelph, Guelph, ON N1G 2W1 Canada

**Keywords:** Canine, Atlantoaxial joint, Atlantoaxial surgery, Computed tomography, Three-dimensional, Neurosurgical methods

## Abstract

**Background:**

Canine ventral atlantoaxial (AA) stabilization is most commonly performed in very small dogs and is technically challenging due to extremely narrow bone corridors. Multiple implantation sites have been suggested but detailed anatomical studies investigating these sites are lacking and therefore current surgical guidelines are based upon approximate anatomical landmarks. In order to study AA optimal safe implantation corridors (OSICs), we developed a method based on computed tomography (CT) and semi-automated three-dimensional (3D) mathematical modelling using OsiriX™ and Microsoft®Excel software. The objectives of this study were 1- to provide a detailed description of the bone corridor analysis method and 2- to assess the reproducibility of the method. CT images of the craniocervical junction were prospectively obtained in 27 dogs and our method of OSIC analysis was applied in all dogs. For each dog, 13 optimal implant sites were simulated via geometrical simplification of the bone corridors. Each implant 3D position was then defined with respect to anatomical axes using 2 projected angles (ProjA). The safety margins around each implant were also estimated with angles (SafA) measured in 4 orthogonal directions. A sample of 12 simulated implants was randomly selected and each mathematically calculated angle was compared to direct measurements obtained within OsiriX™ from 2 observers repeated twice. The landmarks simulating anatomical axes were also positioned 4 times to determine their effect on ProjA reproducibility.

**Results:**

OsiriX could be used successfully to simulate optimal implant positions in all cases. There was excellent agreement between the calculated and measured values for both ProjA (ρ_c_ = 0.9986) and SafA (ρ_c_ = 0.9996). Absolute differences between calculated and measured values were respectively [ProjA = 0.44 ± 0.53°; SafA = 0.27 ± 0.25°] and [ProjA = 0.26 ± 0.21°; SafA = 0.18 ± 0.18°] for each observer. The 95 % tolerance interval comparing ProjA obtained with 4 different sets of anatomical axis landmarks was [−1.62°, 1.61°] which was considered appropriate for clinical use.

**Conclusions:**

A new method for determination of optimal implant placement is provided. Semi-automated calculation of optimal implant 3D positions could be further developed to facilitate preoperative planning and to generate large descriptive anatomical datasets.

**Electronic supplementary material:**

The online version of this article (doi:10.1186/s12917-016-0824-3) contains supplementary material, which is available to authorized users.

## Background

Canine AA instability (AAI) has been treated via surgical stabilization for almost 50 years [1]. Methods of stabilization have evolved from simple dorsal AA sutures to ventral transarticular screw fixation (TSF) and more complex constructs composed of multiple ventral implants embedded in polymethylmethacrylate cement [2–6]. Despite overall satisfactory outcomes obtained with modern procedures [6–8], ventral AA stabilization remains technically challenging and is associated with relatively high mortality rates (5 %) [8].

The small size of affected dogs and extremely narrow bone corridors used to position stabilizing implants are often considered major technical limitations of these procedures [9, 10]. These technical difficulties have led neurosurgeons to develop novel techniques to either improve accuracy of implant placement or to multiply the number of implants to better distribute the load applied on the stabilizing construct [2–6, 9, 10]. Complications directly resulting from such narrow bone corridors include iatrogenic bone fracture and violation of the vertebral canal by the stabilizing implants. Both of these complications can have disastrous consequences for the patient either by compromising the stability of the construct or by causing iatrogenic spinal cord injury. Although the incidence of either complication in canine AA stabilization is unknown, experimental studies on ventral placement of pedicular and monocortical implants in other cervical vertebrae have demonstrated that vertebral canal violation is common [11, 12]. It can be hypothesized that vertebral canal violation in AA stabilization is likely as common and underestimated by clinicians given that radiographs have a low sensitivity to detect vertebral canal violation and that postoperative CT is not commonly performed in veterinary medicine [13].

In human medicine, extensive precautions are taken to avoid both vertebral canal violation and vertebral artery injury when performing cervical and AA stabilization. Routine stabilization procedures rely on readily available anatomical data defining OSICs, preoperative planning using advanced imaging and various imaging-based intraoperative guidance techniques [14–19]. In veterinary spinal surgery, preoperative planning is often limited and intraoperative neuronavigation is not routinely available, leaving neurosurgeons with the descriptive data provided by the literature to guide implant placement.

Traditionally, vertebral OSICs have been characterized using radiographic and CT images. This has been achieved by identifying a theoretical plane within which the optimal implant is to be positioned. Using such a predefined plane allows simplification of a complex 3D problem into a bidimensional description. This method has been applied successfully along most of the vertebral column because stabilizing implants are typically positioned within the transverse plane of each vertebra [20–23]. However, most of the reported atlas (C1) and axis (C2) implantation sites have an oblique direction precluding the use of these traditional methods. As a result, AA implant sites have been subjectively defined without detailed anatomical description. The only OSIC that has been more precisely studied in dogs is the corridor used for TSF fixation [5, 9]. However, available studies have used different subjective definitions of the optimal implant position and therefore obtained slightly different results. Overall, precise objective surgical guidelines are currently lacking for AA ventral stabilization.

Our initial objective was to provide data to the veterinary community describing as precisely as possible optimized methods of AA stabilization. As we began to work on the description of optimal implant positioning, we realized that a new method of analysis of bone corridors would be necessary to precisely depict the complex 3D interrelationships between anatomical structures and implants. Therefore, we planned to develop a novel approach for the analysis of CT 3D data, using the Digital Imaging and Communication in Medicine (DICOM) software OsiriX™. In order to apply this method in a time-efficient manner, a mathematical model was also developed to semi-automatize the process.

In the present study, our objective was to describe and validate this new method of bone corridor analysis. We hypothesized 1- that mathematical calculations would have high concordance when compared to manual measures, and 2- that predefined anatomical landmarks could be used reliably to calculate 3D implant coordinates.

## Methods

### Preliminary review of the literature

An extensive online literature search was conducted prior to the study in order to identify available descriptions of canine AA anatomy as well as bone corridors previously used for ventral AA stabilization. Both PubMed and Google scholar search engines were used to identify pertinent publications with the search terms “ventral atlantoaxial dog”, “atlantoaxial instability dog” or “atlantoaxial subluxation dog”. When using Google scholar, only the first 250 references were assessed for relevance. Articles written in a language other than English were excluded. All identified journal articles and textbook chapters describing AA anatomy or ventral AA stabilization techniques were reviewed in detail, including identification of pertinent cited references which were also reviewed.

### Study population and CT image acquisition

Between October 2012 and December 2013, dogs were prospectively recruited in order to obtain a CT scan of their craniocervical region. The objective was to recruit approximately 10 dogs affected with AAI and 20 dogs with a normal AA joint (including 10 Toy breed dogs and 10 Beagle dogs). Toy breed dogs were defined as dogs with a body weight less than 5 kg. A minimal age of 6 months was subjectively selected to avoid excessive anatomical variations due to growth stage. The CT scans obtained from dogs with a normal AA joint were either obtained from cadavers or client owned animals anesthetized at the Ontario Veterinary College Health Sciences Centre for clinical reasons unrelated to this study after owner consent was obtained. For AAI dogs, obtaining a CT scan of the AA region is standard-of-care practice for diagnostic workup and/or pre-surgical planning in our institution. A diagnosis of AAI was reached if dens separation, agenesis, or hypoplasia was identified in conjunction with clinical signs consistent with a cranial cervical myelopathy or if unequivocal AA subluxation was visible in the CT study.

CT images of the craniocervical junction were obtained using a 16 slice detector GE Brightspeed CT scanner.^1^ The raw data was acquired with a standardized protocol in helical mode, 1.0 s rotation time, 0.562:1 pitch, 120 kV and 250mAs, 25 cm collimation, 512x512 matrix size, 0.488 mm in plane resolution, 0.625 mm through plane resolution using both standard and bone algorithms. Both algorithms were reviewed but only the images captured in a bone algorithm were used for OSIC analysis. The images were subsequently imported into the free version of OsiriX™ DICOM viewer^2^ using an Apple® computer.^3^ The images were reviewed using the window width and level preset in OsiriX™ for bone CT images in the 2D viewer, 3D multi-planar reconstruction (MPR) and 3D volume rendering (VR) modes.

### 3D optimal implant simulation using OsiriX™

**Fig. 1:**
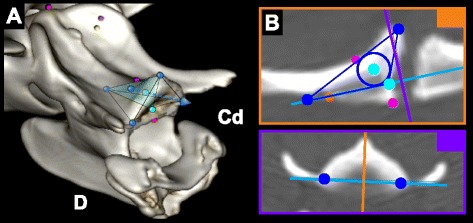
Principles of determination of optimal implant placement using the geometrically centered method. **a** Example of simplification of a bone corridor into a pyramidal shape (*theoretical black lines*) using ROI points (*in blue*) placed in 3D-VR mode. An optimal implant can be positioned using an axis centered within the pyramid (blue dashed line). **b** This centered axis is determined in 3D-MPR mode using ROI points (*in blue*) as landmarks and geometrical shapes (*dark blue lines*) [see Additional file 1 for more details]. D: dorsal; Cd: caudal. ROI points in other colors correspond to different implant sites

In order to define objective optimal implant placements for each available bone corridor, we developed a method based on geometrical simplification of the bone corridors. The purpose of this simplification was to obtain 3D geometrical shapes with well-defined centered axes, that could then be used to define optimal implant placements. To be surgically applicable, the insertion point for each implant had to be located on the ventral surface of C1 or C2. The geometrical shapes simulating the bone corridors were delineated in OsiriX™ by placing region of interest (ROI) points either in 3D-MPR mode or 3D-VR mode. These ROI points were subsequently used as landmarks in 3D-MPR mode to determine centered axes of the bone corridors using various geometrical methods (Fig. 1). Each optimal implant placement could then be simulated by placing 2 ROI points along the centered axis representing the insertion and exit points of the implant. Further details on how each specific bone corridor was geometrically simplified and each optimal implant placement was obtained is provided in additional files (see Additional file 1).

### Determination of optimal implant 3D coordinates by manual measurements

**Fig. 2:**
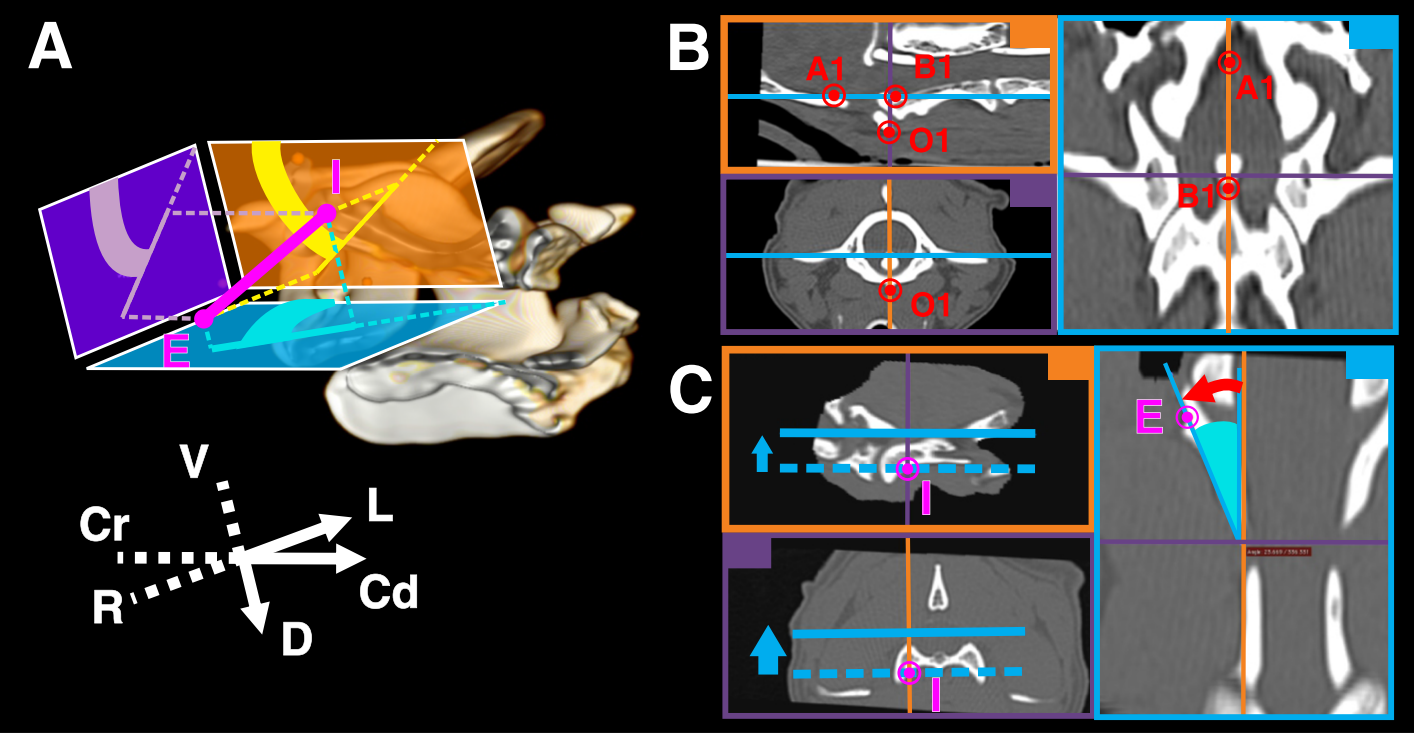
Manual measurement of ProjA using OsiriX™. **a** Sagittal, transverse and dorsal ProjA can be used as the implant 3D coordinates. **b** To determine ProjA using OsiriX™ 3D-MPR mode, the 3 planes are first aligned with the anatomical planes (points OAB are used as landmarks – see Fig. 4). **c** ProjA measurement is obtained by shifting the plane of projection from the insertion point (I) to the exit point (E) of the studied implant (this is an example of dorsal ProjA). V: ventral; D: dorsal; R: right; L: left; Cr: cranial; Cd: caudal

Each implant direction was defined using 2 ProjA on anatomical planes, which were used as 3D coordinates (Fig. 2a). It is important to note that projections onto anatomical planes are not identical between C1 and C2 because of the significant range of motion existing between these vertebrae. In this study, a projection on C1 anatomical planes was operated for C1 implants and respectively for C2 implants. Transarticular implants involving C1 and C2 were defined using C1 projections. OsiriX™ allowed determination of the ProjA by aligning 3D-MPR planes with anatomical planes. This was achieved by identifying 1 anatomical plane and 1 anatomical axis. For both C1 and C2, the sagittal plane was defined as the plane of symmetry of the vertebra which was determined in 3D-MPR mode. Then, an anatomical axis was identified in the sagittal plane to complete the alignment of all 3 planes. For C1, the ventrodorsal axis was defined as the sagittal cranial border of C1 dorsal and ventral arches. For C2, the craniocaudal axis was defined as the sagittal ventral border of the C2 vertebral foramen (Fig. 2b). Once anatomical alignment was obtained in 3D-MPR mode, ProjA could be measured manually by centering the intersection of the 3 planes on the insertion point followed by shifting the plane of interest until the exit point was visualized (Fig. 2c).

### Estimation of the safety margins of each OSIC by manual measurements

**Fig. 3:**
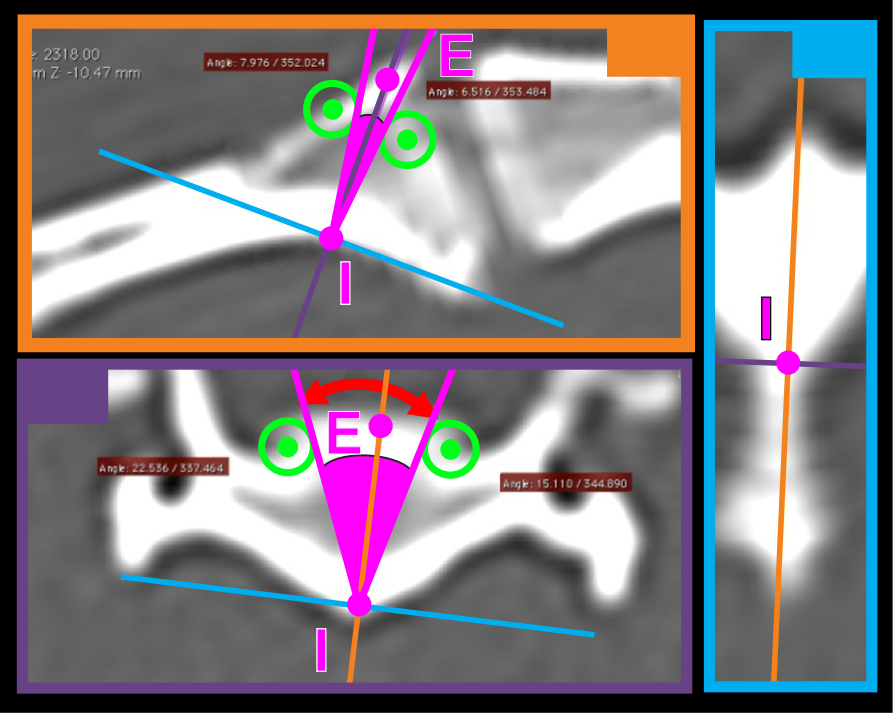
Manual method of determination of SafA using OsiriX™ 3D-MPR mode. First, 2 orthogonal planes containing the safety margin points (*green points*) are identified. The intersection of these 2 planes simulates the implant site (IE). Then, circles of the same diameter as the simulated implant are positioned around the safety point (*green circles*). Tangent lines to each circle passing through the insertion point are used to measure SafA (*red arrows*)

In order to provide an estimation of the safety margins associated with each implant site, we developed a new method considering both the bone margins and the diameter of the implant used. The general principle of the method was to study the bone corridor associated with each optimal implant site in 2 subjectively defined orthogonal planes using OsiriX™ 3D-MPR mode. In each of these planes, 2 safety margins were determined by rotating the central axis of the implant away from the optimal position around the insertion point until the implant position became considered unsafe (either due to inappropriate bone purchase or violation of vital structures). Bone purchase was considered inappropriate when the virtual implant central axis reached a line tangent to the inner surface of the near (cis) vertebral cortex. Vital structures included the spinal cord, nerve roots or blood vessels passing through or in between C1 and C2. These structures were delineated in CT images by the vertebral, lateral, alar, transverse and intervertebral foramen. The first point encountered along each rotation of the implant that was causing it to become unsafe was identified by placing an ROI point representing a safety margin of the bone corridor. For sagittal implants directed toward the vertebral canal, 75 % of the corridor length was used to position safety margin points. This method allowed identification of 4 safety margin points for each bone corridor. SafA could then be determined using the optimal implant ROI points, the safety margin ROI points and circles simulating the implant diameter as demonstrated in Fig. 3.

### Mathematical determination of optimal implant 3D coordinates and OSIC safety margins

The methods of determination of ProjA and SafA described above were considered excessively time consuming to apply on a large sample size and to be used routinely in the clinical setting. Given that we intended to study multiple implant sites in approximately 30 dogs, we elected to develop a method of mathematical calculation of these angles that would be more time efficient. All mathematical calculations were performed using Microsoft® Excel software.^4^ The calculations were based on the CT 3D coordinates of the ROI points simulating the optimal implants and their respective safety margins. An open-source OsiriX™ plugin^5^ allowing exportation of 3D coordinates into Microsoft® Excel was used to optimize the process and limit the risk of error while transferring the 3D data.

**Fig. 4:**
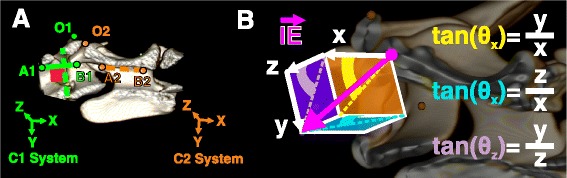
Trigonometric equations used for ProjA calculations based on anatomical coordinates. **a** Anatomical coordinate systems were defined for C1 and C2 by positioning 3 points (O, A, B) in the sagittal plane of each vertebra. The point O represents the origin of the coordinate system, while (AB) represents the craniocaudal axis (X). **b** Geometrical demonstration of sagittal (*yellow*), dorsal (*blue*) and transverse (*purple*) ProjA (Ɵ) calculation based on anatomical coordinates (x, y, z) of a vector $\left(\overrightarrow{\boldsymbol{IE}}\right)$

Mathematical determination of ProjA required advanced vectorial calculations (see Additional file 2 for details). Briefly, each optimal implant was considered as a vector oriented from its insertion to exit point. The coordinates of these points provided by the CT scan could not be directly utilized for ProjA calculations. Instead, a change of coordinate system (also called change of basis) was performed to provide vector coordinates defined with respect to the anatomical axes. This was achieved by defining anatomical coordinate systems for C1 and C2 using 3 ROI points strategically selected in the sagittal plane of each vertebra (Fig. 4a). Once the coordinates of the vector were determined with respect to the anatomical axes, the ProjA could easily be calculated using standard trigonometric equations (Fig. 4b). Each step of the calculations was then entered into a Microsoft® Excel sheet semi-automating the process.

**Fig. 5:**
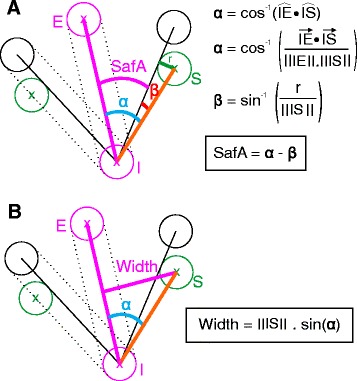
Geometrical demonstration of SafA and OSIC width calculations. **a** Method of SafA calculation; (**b**) Method of OSIC width calculation. In the schematics, the IE segment represents the optimal implant position (*in pink*) and the S point is the safety margin identified on CT images by rotating the axis of the implant around its insertion point until the position is considered unsafe. The insertion point (I), exit point (E), safety margin (S), and radius of the implant (r) are all known entities

Mathematical equations for calculation of the SafA could be established through trigonometry as depicted in Fig. 5a. The CT coordinates of the safety margins ROI points could be used without any change of basis, as these angles are defined with respect to the axis of the optimal implant. The diameter of the implant used had to be known to calculate SafA. The same safety margin ROI points could also be used to estimate the width of the bone corridor in both orthogonal planes as depicted in Fig. 5b. An example of the spreadsheet with all pre-entered equations for all 13 implant sites is provided as an additional file (see Additional file 3).

### Mathematical model validation and estimation of angle measurement errors

In order to validate our semi-automated method of determination of ProjA and SafA, a complete OSIC simulation was performed by 1 author (GL) including simulation of 13 optimal implants (2 ROIs/implant), safety margins (4 ROIs/implant) and C1/C2 anatomical axes (6 ROIs/dog) in all dogs. A sample of 12 dogs was then randomly selected within the recruited population and 1 implant was randomly selected for each dog using random numbers generated with SAS OnlineDoc® software.^6^ Prior to any angle measurements and calculation, the ROI points simulating the selected optimal implant and its associated anatomical coordinate system and safety margins were imported in OsiriX™ for each dog. These ROI points were then used to determine 2 ProjA and 4 SafA mathematically and compared to measures obtained manually for each of the 12 selected implants. The manual measures were obtained by 2 observers (AZ and GL) and repeated after a 1-week interval. For determination of SafA, implant diameter was subjectively set at 1.5 mm. Agreements between the calculated values and manual measures and 95 % tolerance limit intervals were determined to validate the mathematical model.

In order to estimate the error generated by the operator when measuring ProjA and SafA manually, calculated values were considered as gold standard. The measurement error was determined for each observer by calculating the absolute difference between the manually measured and calculated values.

In order to estimate the error on ProjA measurements generated by the operator when identifying the anatomical axes, 6 ROI points representing the anatomical coordinate systems of C1 (O_1_, A_1_, B_1_) and C2 (O_2_, A_2_, B_2_) were positioned by 2 observers (AZ and GL) with 2 repeats in all 12 dogs. This provided 4 sets of 6 ROIs per dog, each representing the same anatomical axes with a slight variation due to operator variability. To estimate the effect of this variability on ProjA values, all previously determined ROI points representing all of the studied implant sites (13/dog) were imported into OsiriX™ for all 12 dogs. For each implant site, 2 ProjA were calculated 4 times based on the 4 coordinate systems obtained by the 2 observers. Because the exact position of the anatomical axes cannot be objectively determined, the mean of the 4 repeats of each ProjA values was used as gold standard for that part of the study. Agreements and absolute error between each of the 4 obtained values and the gold standard as well as 95 % tolerance limit intervals were determined to estimate the ProjA calculation error. Agreements and 95 % tolerance limit intervals were also determined between observers, between repeats and within subsamples (specific coordinate systems or specific projection planes) in order to identify the most significant sources of error.

### Statistical analysis

Agreements between different methods of angle determination (automatically calculated vs manual) and between repeated measures were obtained using the Bland-Altman method and concordance correlation. This method also allowed calculation of 95 % tolerance intervals, representing the range of values that would theoretically be obtained for a single measure with 95 % probability. These values estimated the reproducibility of the method which is defined as the degree to which repeated measurements provide similar results [24]. Statistical analysis of the data was performed using statistical software SAS OnlineDoc®. Statistical significance was set at a maximum p value of 0.05.

## Results

### Sampled population and CT images acquisition

Over the recruitment period, 27 dogs were recruited to participate in this study. The recruited population differed slightly from the initial objective and included 9 mature Beagle dog cadavers, 13 Toy breed dogs with a normal AA joint and 5 Toy breed dogs affected with AAI. One unaffected Toy breed dog was 3 weeks younger than our inclusion criteria but was not excluded given the CT images revealed complete fusion of the vertebral growth plates suggesting the AA region had reached adult stage. CT images of the craniocervical junction were successfully obtained in 25 dogs using the pre-established protocol. In 2 AAI dogs, the slice thickness was set at 1.25 mm instead of 0.625 mm due to a protocol error.

### Definition of AA OSICs and simulation of optimal implant 3D positions

**Fig. 6:**
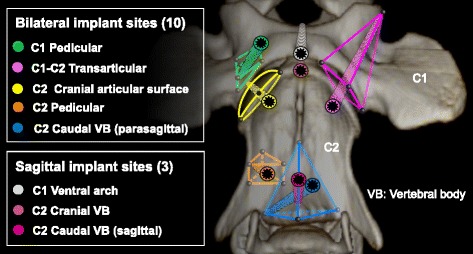
Simulation of 13 optimal implants using geometrical simplification of AA bone corridors (*ventral view*)

The online search of the literature identified 32 pertinent references [4–7, 9, 10, 22, 25–49]. Upon review of these references, 9 safe bone corridors were defined corresponding to anatomical parts of the AA vertebrae. These bone corridors included the lateral masses (C1, bilateral), ventral arch (C1, sagittal), cranial articular surfaces (C2, bilateral), cranial vertebral body (C2, sagittal), pedicles (C2, bilateral) and caudal vertebral body (C2, sagittal). Each corridor was simplified into a geometrical shape, including pyramids, prisms and hemi-ellipsoids (Fig. 6). The general principle used to simulate optimal implants was to identify well-defined centered axes of the geometrical shapes (see Additional file 1). In addition, a transarticular optimal implant position was defined (C1-C2, bilateral) using the lateral mass corridors to define its axis and the ventral surface of C2 to position its insertion point. For the caudal vertebral body corridor, 3 different centered implant positions could be defined. Therefore in total, 13 optimal implant sites could be objectively defined (Fig. 6). For implants located in the sagittal plane, a traditional method was used by identifying 1 point of the optimal axis within the sagittal plane which was then used in 3D-MPR mode to center the implant axis (see Additional file 1).

The method of OSIC analysis was used successfully in all 27 cases although some limitations were observed while positioning ROI points. Even though OsiriX™ 3D modes generated continuous 3D space, ROI points placed in 3D-MPR or 3D-VR modes remained associated to specific slices. In other words, the space located between each slice could not be represented using ROI points. Another seemingly random difficulty encountered in 3D-VR mode was an occasional software glitch when placing ROI points. Instead of positioning the point on the visible bone surface, the point would be placed on the opposite side of the vertebra. This malfunction could be overcome by positioning the vertebra so that the bone surface of interest was tangent to the operator view.

### Validation of the mathematical model and estimation of measurement errors

The raw data of manually measured and calculated values from the 2 observers is presented in Table 1. The 2 CT studies that had 1.25 mm slice thickness (instead of 0.625 mm) were excluded from the random sampling process. Sagittal implants were also excluded due to only 1 projected angle value defining them.

**Table 1:** Mathematically calculated and manually measured values of ProjA and SafA

Case	Implant	Observer	Repeat	ProjA1 (°)	ProjA2 (°)	SafA1 (°)	SafA2 (°)	SafA3 (°)	SafA4 (°)
7	8	1	1	35.67	20.60	16.33	11.54	17.52	5.60
		1	2	34.59	22.66	15.9	11.9	17.42	5.65
		2	1	34.72	21.36	16.48	11.29	17.46	5.41
		2	2	34.39	21.43	16.59	11.16	17.44	5.51
		*Gold standard*		**34.51**	**21.42**	**16.50**	**11.25**	**17.38**	**5.51**
3	3	1	1	35.78	33.80	13.38	10.29	4.02	4.58
		1	2	35.69	34.34	13.01	9.99	4.51	4.49
		2	1	35.21	33.77	13.02	10.51	4.19	4.46
		2	2	35.60	34.52	13.45	10.27	4.23	4.39
		*Gold standard*		**35.61**	**34.30**	**13.20**	**10.19**	**4.15**	**4.44**
22	1	1	1	25.67	23.66	44.28	12.71	7.68	9.30
		1	2	25.63	24.04	44.54	13.38	7.75	9.31
		2	1	25.39	24.06	44.23	13.34	7.35	9.34
		2	2	25.28	23.96	44.76	13.50	7.37	9.42
		*Gold standard*		**25.61**	**24.10**	**44.38**	**13.21**	**7.55**	**9.29**
18	4	1	1	41.14	37.39	24.12	21.55	40.16	15.95
		1	2	43.13	37.12	23.38	21.88	41.39	14.61
		2	1	43.29	38.21	23.72	21.56	40.87	15.69
		2	2	42.97	37.94	24.39	20.94	40.52	15.74
		*Gold standard*		**42.81**	**38.27**	**23.54**	**21.63**	**40.65**	**15.83**
15	0	1	1	30.03	16.73	44.61	11.91	16.48	6.62
		1	2	30.3	19.61	45.86	10.82	16.29	6.25
		2	1	30.32	19.99	45.61	10.72	16.29	5.63
		2	2	30.00	19.42	45.57	10.69	16.46	6.04
		*Gold standard*		**29.83**	**19.68**	**45.64**	**10.72**	**16.17**	**6.30**
25	8	1	1	26.09	14.03	17.62	19.69	13.60	3.29
		1	2	26.13	15.66	17.17	19.51	13.69	3.71
		2	1	26.09	15.36	16.83	19.86	13.88	3.53
		2	2	25.93	15.26	17.88	19.72	13.91	3.40
		*Gold standard*		**26.08**	**14.93**	**17.35**	**19.85**	**13.81**	**3.41**
14	7	1	1	7.22	31.70	31.87	15.80	28.14	19.85
		1	2	8.18	31.95	31.35	15.5	27.73	19.16
		2	1	6.99	32.51	31.19	15.04	27.42	18.81
		2	2	6.90	31.16	31.37	15.46	26.99	19.56
		*Gold standard*		**7.30**	**31.62**	**31.21**	**15.56**	**27.51**	**19.21**
16	2	1	1	32.00	35.50	11.11	17.58	13.84	3.58
		1	2	31.94	35.41	11.27	17.18	13.49	3.51
		2	1	31.33	35.88	11.17	17.17	13.77	3.34
		2	2	31.32	35.22	11.07	17.03	13.76	3.40
		*Gold standard*		**31.33**	**35.34**	**11.21**	**17.09**	**13.74**	**3.41**
20	2	1	1	25.62	22.77	14.24	22.02	9.19	2.45
		1	2	25.82	22.91	14.15	21.81	8.81	2.31
		2	1	25.65	23.38	14.28	21.86	9.08	2.08
		2	2	25.69	23.27	14.14	21.73	8.87	2.25
		*Gold standard*		**25.61**	**23.13**	**14.23**	**21.81**	**9.08**	**2.17**
5	8	1	1	39.27	11.33	9.48	8.74	18.57	0.74
		1	2	39.25	10.63	9.12	8.79	18.57	0.45
		2	1	38.73	11.71	9.35	8.39	18.74	0.47
		2	2	39.00	11.41	9.04	8.55	18.89	0.43
		*Gold standard*		**38.98**	**11.06**	**9.22**	**8.60**	**18.80**	**0.38**
24	7	1	1	2.96	22.15	34.91	26.15	29.58	27.10
		1	2	2.34	22.61	33.99	26.9	30	26.41
		2	1	1.59	22.94	33.46	25.97	29.66	26.49
		2	2	2.19	22.25	34.04	26.72	29.27	27.36
		*Gold standard*		**2.45**	**22.71**	**33.96**	**26.54**	**29.32**	**26.95**
2	0	1	1	15.71	20.17	45.65	11.82	11.85	11.69
		1	2	15.32	20.35	46.05	12.23	12	12.35
		2	1	15.63	20.10	45.29	11.81	11.34	11.61
		2	2	15.30	19.58	45.54	12.22	11.92	11.21
		*Gold standard*		**15.49**	**20.00**	**45.66**	**12.04**	**11.97**	**11.48**

Implants were numbered as follows: C1 pedicular (0–1); C1-C2 transarticular (2–3); C2 cranial articular surface (4–5); C2 pedicular (6–7); C2 parasagittal caudal vertebral body (8–9); Right side (even #); Left side (odd #); Bold font: Mathematically calculated values (gold standard)

**Fig. 7:**
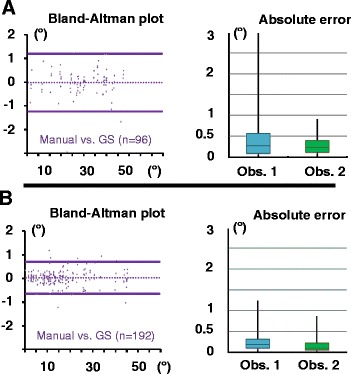
Graphical representation of manual measurement reproducibility and absolute operator-induced error. **a** Bland-Altman and 4 quartiles box plots of absolute errors on ProjA values. **b** Bland-Altman and 4 quartiles box plots of absolute errors on SafA values. Bland-Altman plots are used to represent the difference between each measurement and the gold standard (GS) on the y axis. If perfect agreement between the 2 compared methods was present, all the points would be located on the 0 line. The 2 lines parallel to the 0 line represent the 95 % tolerance limits which is the expected error on a single measurement with respect to the gold standard with 95 % probablity

Excellent agreement was observed between the calculated and measured values for both ProjA (ρ_c_ = 0.9986) and SafA (ρ_c_ = 0.9996). The 95 % tolerance intervals obtained by concordance analysis to estimate operator-induced error for manual angle measurements by comparison to semi-automated calculations (gold standard) were respectively, [−1.23°,1.20°] and [−0.65°,0.70°] for ProjA and SafA. Absolute errors were, respectively, [ProjA = 0.44 ± 0.53°; SafA = 0.27 ± 0.25°] and [ProjA = 0.26 ± 0.21°; SafA = 0.18 ± 0.18°], for each observer. Results from this concordance analysis and measurement errors are summarized in Tables 2 and 3, and graphically represented in Fig. 7. These results implied that our mathematical model was in agreement with manual measures and that manual measurement of these angles was very reproducible with minimal operator-induced error.

**Table 2:** Concordance analyses validating our mathematical method and estimating error generated by manual measures

Angle	Value 1	Value 2	n	R^2^	ρ_c_	Low TL	Up TL	Low CI	Up CI	Bias (°)	*P*-value
ProjA	**GS**	**Single value**	96	0.9972	**0.9986**	**−1.23**	**1.20**	−0.13	0.09	−0.019	0.726
	Rep1	Rep2	48	0.9944	0.9971	−1.94	1.75	−0.32	0.13	−0.092	0.415
	Obs1	Obs2	48	0.9937	0.9968	−1.99	1.92	−0.27	0.20	−0.033	0.779
SafA	**GS**	**Single value**	192	0.9992	**0.9996**	**−0.65**	**0.70**	−0.02	0.07	0.027	0.239
	Rep1	Rep2	96	0.9984	0.9992	−1.04	1.00	−0.11	0.07	−0.020	0.677
	Obs1	Obs2	96	0.9983	0.9991	−0.94	1.17	0.02	0.21	0.116^a^	0.019

^*a*^statistically significant bias (considered clinically non-significant); Bold font: agreement between GS and manually measured values

*n*: number of measures compared, *R* ^*2*^ correlation coefficient, *ρ*_*c*_ concordance coefficient, *TL* 95 % tolerance interval limit (in degrees), *CI* 95 % confidence interval limits (in degrees), *GS* gold standard (mathematically calculated values), *Obs* observer, *Rep* repeat

**Table 3:** Absolute errors determined by comparison between manual and mathematical values

Angle	Repeats	Observer 1	Observer 2
ProjA	1	0.55 ± 0.65	0.31 ± 0.25
	2	0.34 ± 0.35	0.21 ± 0.14
	Both	0.44 ± 0.53	0.26 ± 0.21
Saf A	1	0.29 ± 0.26	0.18 ± 0.18
	2	0.24 ± 0.25	0.17 ± 0.18
	Both	0.27 ± 0.25	0.18 ± 0.18

Values (in degrees) reported as Mean ± Standard deviation

### Validation of the anatomical axes simulation and estimation of the error induced by landmark placements

**Fig. 8:**
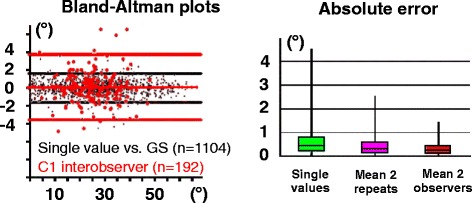
Graphical representation of OAB landmarks reproducibility and absolute operator-induced error on ProjA calculations. Two Bland-Altman plots are superimposed including the overall comparison between the gold standard (GS) and single ProjA calculated values (*in black*) and the widest observed 95 % tolerance limit interval comparing values from 2 different observers in C1 system subsample (*in red*)

The raw data of ProjA values calculated based on 4 sets of anatomical landmarks representing C1 and C2 coordinate systems is presented in the additional files (see Additional file 4). Agreements between each value and the gold standard (mean of 4 values) revealed to be excellent (ρ_c_ = 0.9985) with an overall low 95 % tolerance interval [−1.62°, 1.61°]. The absolute error (mean ± SD) was determined by comparing the gold standard to each individual value (0.58 ± 0.54°), to the mean of 2 values from the same observer (0.42 ± 0.39°) and to the mean of 2 values from different observers (0.30 ± 0.25°). Agreement analysis within subsamples including coordinate system (C1 or C2) and the plane of projection used (Sagittal/Transverse/Dorsal) was conducted between observers and between repeats. This revealed that the widest 95 % tolerance interval was obtained when comparing inter-observer values calculated in C1 coordinate system [−3.58°, 3.72°]. Results from this concordance analysis and calculated errors are summarized in Tables 4 and 5, and graphically represented in Fig. 8. These results suggest that the simulation of anatomical axes in OsiriX™ using the previously defined OAB points is very reproducible. The largest predicted error on an individual ProjA value was estimated at 3.7° (with 95 % probability).

**Table 4:** Concordance analyses estimating the reproducibility and error generated by OAB landmark placements

Angle	Value 1	Value 2	n	R^2^	ρ_c_	Low TL	Up TL	Low CI	Up CI	Bias (°)	*P*-value
ProjA	**GS**	**Single value**	1104	0.9970	**0.9985**	**−1.62**	**1.61**	−0.05	0.04	−0.006	0.801
	**GS**	**Mean 2 Obs**	552	0.9992	**0.9996**	**−0.83**	**0.83**	−0.04	0.03	−0.003	0.861
	Obs1	Obs2	552	0.9912	0.9956	−2.85	2.79	−0.15	0.08	−0.032	0.579
	**Obs1-Sag**	**Obs2-Sag**	264	0.9909	**0.9955**	**−3.37**	**3.25**	−0.25	0.13	−0.060	0.533
	Obs1-Tr	Obs2-Tr	144	0.9815	0.9905	−2.02	2.27	−0.04	0.29	0.123	0.135
	Obs1-Dors	Obs2-Dors	144	0.9844	0.9921	−2.96	2.69	−0.35	0.08	−0.137	0.207
	**Obs1-C1**	**Obs2-C1**	192	0.9621	**0.9808**	**−3.58**	**3.72**	−0.17	0.32	0.074	0.549
	Obs1-C2	Obs2-C2	360	0.5493	0.9976	−2.48	2.31	−0.21	0.03	−0.089	0.142
	**GS**	**Mean 2 Rep**	552	0.9984	**0.9992**	**−1.18**	**1.18**	−0.05	0.05	−0.001	0.957
	Rep1	Rep2	552	0.9943	0.9971	−2.33	2.20	−0.16	0.03	−0.065	0.163
	Rep1-Sag	Rep2-Sag	264	0.9941	0.9970	−2.77	2.58	−0.25	0.06	−0.098	0.211
	Rep1-Tr	Rep2-Tr	144	0.9846	0.9922	−1.97	1.95	−0.16	0.14	−0.008	0.916
	Rep1-Dors	Rep2-Dors	144	0.9918	0.9959	−2.11	1.98	−0.22	0.09	−0.063	0.421
	Rep1-C1	Rep2-C1	192	0.9795	0.9890	−2.92	2.56	−0.36	0.00	−0.179	0.054
	Rep1-C2	Rep2-C2	360	0.9965	0.9983	−2.06	2.05	−0.11	0.10	0.930^a^	−0.005

Bold font: most clinically relevant concordance analyses including comparisons with GS and widest tolerance limit intervals

*n* number of measures compared, *R* ^*2*^ correlation coefficient, *ρ*_*c*_ concordance coefficient, *TL* 95 % tolerance interval limit (in degrees), *CI* 95 % confidence interval limits (in degrees), *GS* gold standard (mean of 4 values), *Obs* observer, *Rep* repeat, *Sag* sagittal ProjA, *Tr* transverse ProjA, *Dors* dorsal ProjA, *C1* angle projected in C1 system, *C2* angle projected in C2 system

^a^statistically significant bias (considered clinically non-significant)

**Table 5:** Absolute errors determined by comparison between the gold standard and individual values or the mean of 2 values

Angle	Repeats	Observer 1	Observer 2	Mean 2 Observers
ProjA	1	0.63 ± 0.59	0.54 ± 0.60	0.30 ± 0.25
	2	0.60 ± 0.50	0.56 ± 0.44	
	Mean 2 Repeats	0.42 ± 0.39		Overall single values 0.58 ± 0.54

Values (in degrees) reported as Mean ± Standard deviation

## Discussion

This study provides a detailed description of a new method of AA OSIC analysis using OsiriX™. This method overcame the problem of subjective optimal implant placement definitions. Three-dimensional simplification of bone corridors into geometrical shapes not only permitted the description of corridors of complex / oblique distribution, but also to objectively localize the optimal implant position in space. For the transarticular OSIC, the geometrical determination of the corridor centered axis was based on the C1 lateral masses while the insertion point was located on the C2 cranial articular surface. Some occasional issues were encountered when placing ROI points in 3D-VR mode in some cases but they did not preclude successful OSIC simulation in any case. This step was the most time consuming and could not be automatized. Improvement in 3D surgical planning software would be necessary to allow such automation.

For the determination of numerical values describing 3D optimal implant placements and bone corridor characteristics, we developed a semi-automated procedure relying on the 3D coordinates of pre-identified ROI points. This study demonstrated excellent concordance between the semi-automated mathematical calculations and manually measured values which validated our first hypothesis. Our data also implied that SafA and ProjA could be accurately obtained using OsiriX™ measurement tools, although this would be more time consuming compared to the semi-automated method. Similar concordance analysis revealed that positioning of the landmarks used for anatomical space modeling induced low errors (overall 1.6°, up to 3.7° for some subsamples with 95 % probability). It should be emphasized that this source of error is inherent to the use of ProjA values as 3D coordinates. Similar limitations are encountered when using neuronavigation based on fiducial markers [50]. Based on our results, landmark induced error can be significantly reduced if 2 observers position the ROI points successively and the mean of the 2 obtained ProjA values is used instead of an individual value. Regardless, the observed range of error of only a few degrees was considered small given that a minimum 15–20° bone corridor angular width would likely be necessary to recognize a corridor as acceptably safe. A theoretical error in any ROI positioning was expected of up to half the CT study slice thickness (0.3125 mm) which would also have a low impact on most of the OSIC calculated values. This type of error could have been minimized by reformatting all CT studies to 0.1 mm slice thickness.

Overall, these results validated our second hypothesis, allowing us to conclude that the use of OsiriX™ with implementation of mathematical equations on exported 3D coordinates was an efficient and reproducible tool which could be applied on a larger scale to describe AA OSICs. The major advantage of the described method is it can generate 3D data defined with respect to anatomical coordinates. Such data could be used for applications beyond OSIC descriptions. For instance, 3D data can be used to study the biomechanics of complex motions between vertebral motion units or to characterize pathological range of motion such as observed in AAI. Another possible application of this type of analysis would be to develop software able to automatically reduce AA subluxation at the planning stage. This type of 3D anatomical realignment would be extremely helpful to optimize implant positioning for each individual patient and compare different stabilization constructs such as plating systems or other customized implants in virtual 3D space.

These clinical applications all heavily rely on the definition of anatomical coordinate systems. Consequently, a good understanding of these definitions and how angle projections are made is essential. Identification of the sagittal plane is the most intuitive step to define a ProjA as it is also the plane of symmetry of the vertebra. An anatomical axis is then needed within that plane to define a proper coordinate system. In a previous report, the craniocaudal axis for C2 was defined as the ventral border of the vertebral foramen, which is what was also used in our study [9]. To our knowledge, a similar axis had not yet been described for C1. Therefore, we subjectively defined the cranial border of the dorsal and ventral arches as representing the ventrodorsal axis. The implicit assumption was that these C1 and C2 anatomical axes would have good alignment when the AA joint is placed in neutral position. Further investigation on the neutral position of the AA joint would be necessary to assess the accuracy of that assumption. Misalignment of the anatomical axes could result in clinically significant consequences when placing C1 implants. Indeed, only a very small portion of C1 can be visualized intraoperatively which means that estimation of C1 implant 3D position in surgery is mostly based on landmarks present on the ventral surface of C2. Fortunately, this type of misalignment would have limited impact on the risk of vertebral canal violation (except for a C1 ventral arch implant) as it would only affect the implant direction along the sagittal plane, not lateromedially.

One significant limitation in our study was the small number of recruited dog suffering from AAI, the population of clinical interest. However, most implant positions were simulated using the exact same protocol in all 3 groups. One notable difference in methodology in the AAI group was the fact that the insertion point of transarticular implants normally placed on C2 ventral surface had to be approximated due to AA misalignment. In contrast AA misalignment did not affect transarticular implant directions nor their ProjA values as they were solely defined using C1 landmarks (independently from C2 position). Overall, the low number of AAI cases could affect certain descriptive values such as the estimated length of transarticular implants but was not expected to have any significant impact on the optimal implant direction nor the reproducibility of our method.

Neuronavigation can be defined as a group of techniques designed to target neuroanatomical structures intraoperatively using 3D data obtained via advanced imaging. These methods include frame-based systems where the 3D space is defined with respect to a rigid frame, and frameless systems which require more advanced intraoperative 3D calculations, but allow for greater freedom of movement for the surgeon [50]. In veterinary medicine, neuronavigation represents a promising technical advancement in surgical accuracy, even though it remains cost-prohibitive for most clinical practices. The main alternative to neuronavigation is precise pre-surgical planning. The free open-source DICOM software OsiriX™ was initially developed by a human radiologist to improve multidimensional navigation and display of large datasets generated by advanced imaging modalities such as PET-CT or Cardiac CT [51]. The accuracy and reliability of the software have been validated experimentally and compared to other image analysis programs [52–54]. Good accuracy and low measurement error of the software have been demonstrated which is critical for surgical planning of implant placement with narrow safety margins. OsiriX™ rapidly became a successful 3D navigation tool due to its extremely fast and optimized 3D graphics. More recently, several publications in human medicine have reported its use for fast pre-surgical planning, notably in emergency situations where advanced neuronavigation planning is considered excessively time consuming [53, 55]. In our study, OsiriX™ proved relatively intuitive, and overall easy to use. The method presented here is currently relatively cumbersome due to the necessity to export 3D data in a separate software. However, OsiriX could be complemented with pre-surgical planning tools through the development of plugins incorporating mathematical equations similar to those we used in our study. This would allow generating optimal implant placement definitions considering individual morphological variations of the patient. Precise calculations of such coordinates may improve surgical accuracy; however, this would require the development of intraoperative guiding systems able to use these values to position implants in vivo.

Without such guiding system the precise coordinate values provided by our method would remain difficult to apply to actual patients as intraoperative implant positioning would still rely on the surgeon’s ability to reproduce these values in vivo. Current neuronavigation techniques are difficult to use in the AA region of Toy breed dogs due to the small operation field and limited space available for fiducial markers. Therefore, our group has developed a 3D drill guide device using ProjA values to help the surgeon estimate optimal implant position intraoperatively, although investigation of the accuracy of the device will be necessary. In any case, we expect that the method of determination of OSICs presented here may improve accuracy of implant positioning.

## Conclusion

Individual anatomical variations are becoming more commonly recognized in the canine population, and therefore the need for individualized pre-surgical planning will likely be increasing. Neuronavigation is an appealing solution to this problem, but such advanced technology remains currently inaccessible to most veterinary practices. This study described a novel semi-automated method of OSIC analysis using OsiriX™ DICOM viewer software. Geometrical simplification of bone corridors could be applied in the most commonly reported implant sites used for ventral AA stabilization. The method of calculation of ProjA and SafA was successfully validated by comparing the results to manually measured values. The principles described in our method could be further developed as an interactive preoperative/intraoperative planning tool for individualized surgical planning of spinal fixation.

## Additional files

Additional file 1: Detailed optimal safe implantation corridor method of analysis. Step by step method of geometrical simplification of all studied bone corridors and simulation of associated optimal implant placements. (DOCX 4530 kb) Additional file 2: Cartesian vectorial calculations used to determine projected angles of optimal implants. Step by step demonstration of the mathematical equations used for semi-automation of the method. (DOCX 4598 kb) Additional file 3: Calculation sheet used to analyze the 3D data extracted from ROI points. Equations demonstrated in Additional file 2 are pre-entered in this calculation sheet to automate the calculation process. (XLS 161 kb) Additional file 4: Raw data of projected angle values calculated based on 4 different sets of anatomical landmarks. This data was used to determine the reproducibility of projected angle calculations considering the variability caused by anatomical axes simulation. (XLS 101 kb)

## Abbreviations

3D: Three-dimensional
AA: Atlantoaxial
AAI: Atlantoaxial instability
C1: Atlas
C2: Axis
CT: Computed tomography
DICOM: Digital imaging and communication in medicine
MPR: Multi-planar reconstruction
OSIC(s): Optimal safe implantation corridor(s)
ProjA: Projected angle
ROI(s): Region(s) of interest
SafA: Safety angle
TSF: Transarticular screw fixation
VR: Volume rendering

## Footnotes

1 GE Brightspeed CT scanner, GE Healthcare, Milwaukee, WI, USA.

2 OsiriX™ versions 5.8–6.0, Pixmeo SARL, Bernex, Switzerland.

3 Apple® computer Mac mini, Apple Inc., Cupertino, CA, USA.

4 Microsoft® Excel software version 2011, Microsoft Corp., Redmond, WA, USA.

5 ExportROIs version 1.3.1.

6 SAS OnlineDoc® version 9.2, 2007, SAS Institute Inc. Cary, NC, USA.
