# Supplementary material for: Canine atlantoaxial optimal safe implantation corridors – description and validation of a novel 3D presurgical planning method using OsiriX™
Source: BMC Vet Res. 2016 Sep 6;12(1):188. doi: 10.1186/s12917-016-0824-3 (PMC5012052; doi:10.1186/s12917-016-0824-3)
Supplement: Additional file 1: — Detailed optimal safe implantation corridor method of analysis. Step by step method of geometrical simplification of all studied bone corridors and simulation of associated optimal implant placements. (DOCX 4530 kb) [file 12917_2016_824_MOESM1_ESM.docx]

ADDITIONAL FILE 1: DETAILED OPTIMAL SAFE IMPLANTATION CORRIDOR METHOD OF ANALYSIS

General method of determination of geometrical centers

To determine the center of complex 3D shapes, a specific plane of interest was identified in 3D-MPR mode. This sectioning method of the 3D shape would allow studying simple 2D geometrical objects such as triangles, parallelograms, trapezoids, irregular quadrilaterals, or ellipses. For the purpose of our study, the center of interest of these geometrical shapes could be defined as the point that is the most equidistant from the sides. Such position would theoretically maximize the diameter of the implant that could be inserted into the bone corridor. The method is detailed in Fig A.1.1.

Determination of C1 pedicular optimal implantation axis

The OSIC of the C1 pedicular implants can be defined as the caudal region of C1 lateral masses. We elected to focus on the caudal region of the lateral masses because it is the broadest region and also to avoid penetrating the small artery branching from the vertebral artery toward the vertebral canal at the cranial extremity of the alar foramen. Because of the complexity of the lateral masses’ 3D structure, we elected to define the optimal position, focusing on its narrowest portion at the level of the vertebral artery as well as C1 pedicular region as depicted in Fig A.1.2.


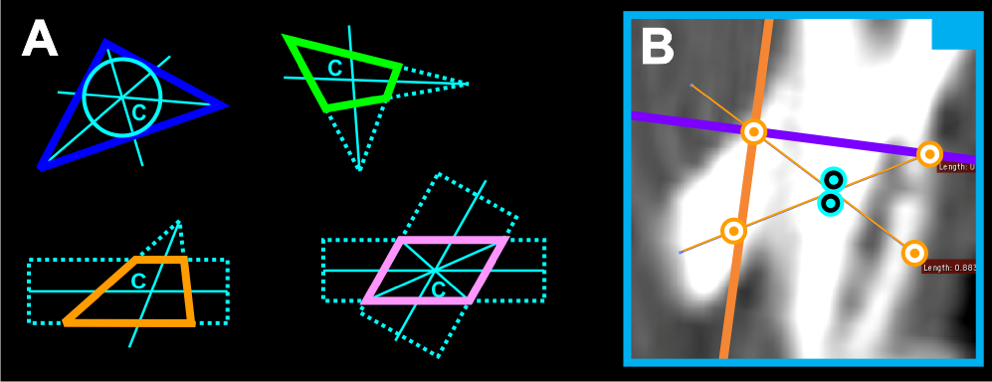


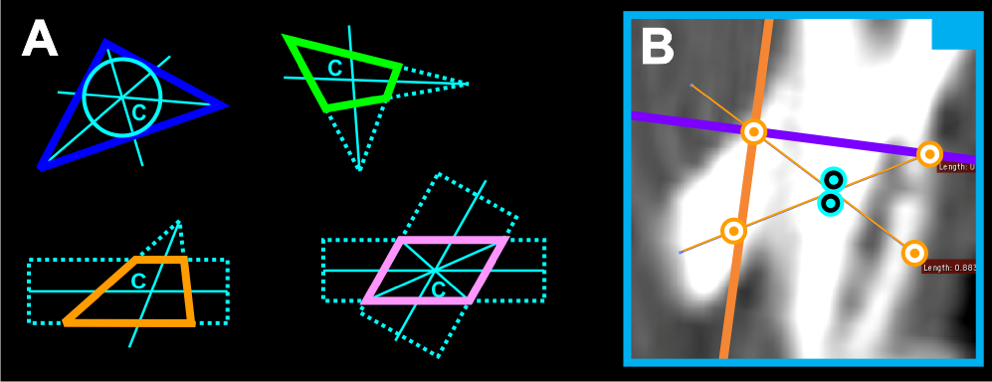


1. Diagrams representing the methods of geometrical determination of OSIC centers.
   (A) For triangles and irregular quadrilaterals, the intersection of 2 bisectors was used to determine the center C. For parallel lines, a bisector line is defined as the equidistant parallel line. This geometrical principle could be used for parallelograms and the parallel lines of a trapezoid. The center of triangles and parallelograms could alternatively be determined using the inscribed circle, and the intersection of diagonals respectively. Centers of ellipses were provided by OsiriX™ ellipse ROI tool. (B) In OsiriX™, the ROI points were associated with specific slices, while centers could be located in between. In these situations, 2 ROI points (in blue) were placed cranially and caudally to delineate the center point as precisely as possible (intersection of orange lines).


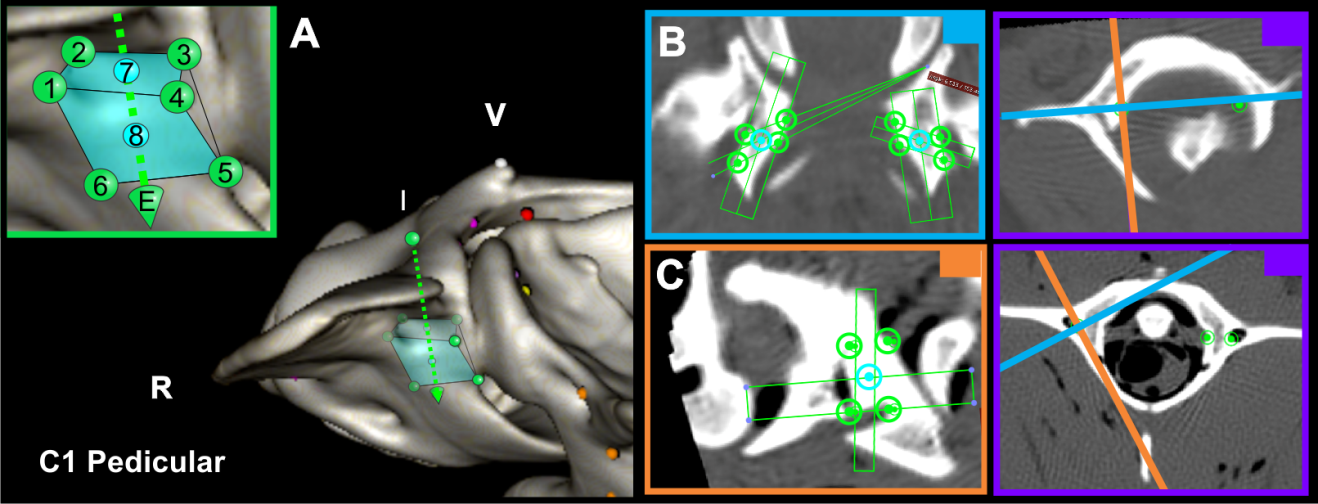


1. Step-by-step method of determination of C1 pedicular optimal implant.
   (A) 3D reconstruction showing C1 pedicle simplified into a prism, (B) Geometrical determination of the center of the ventral surface, (C) Geometrical determination of the center of the lateral surface.
   Point 1 is first positioned in 3D-VR at the craniomedial extremity of the alar foramen (just caudal to the entry point of the tranverse vertebral artery branch). The dorsal plane passing through 1, is localized in MPR-mode. Point 2 (closest point to 1 on the inner cortical) and point 3 (caudomedial extremity) are then positioned. In the same plane, a line tangent to the caudal articular surface of C1 passing through 3 is drawn. The intersection between that line and the lateral cortex localize point 4. Point 5 is defined in 3D-VR as the caudodorsal extremity of the lateral mass. Point 6 is identified in 3D-MPR in the plane 1,4,5 to delineate the pedicle craniodorsal extremity. The centers 7 and 8 are geometrically determined in 3D-MPR. The line 7-8 defines the optimal implant placement.

Determination of C1-C2 transarticular optimal implantation axis

The main challenge of defining C1-C2

transarticular implant position was that 2 separate vertebrae were involved. We elected to define the OSIC based on C1 lateral masses because it is located further away from the implant’s insertion point and contains the longest corridor length (Fig A.1.3). These characteristics suggested C1 was more likely to restrict the optimal implant position compared to C2 cranial articular surface which is a very broad structure. The intent when simplifying the corridor was to describe the region of the lateral masses that would avoid both the vertebral canal and the alar foramen.

Determination of C2 cranial articular surface optimal implantation axis

The cranial articular surfaces of C2 can roughly be approximated to 2 semi-ellipsoids oriented obliquely on each side of the cranial region of the C2 vertebral body. The centered axis of a hemi-ellipsoid can be defined as the intersection of 2 orthogonal bisector planes. However, such a theoretical centered axis would be oriented within the dorsal plane which cannot be achieved when placing implants in practice. Therefore, a subjective angle of 30° from the dorsal plane was used to provide the description of an “optimal” implant that can be used in surgery (Fig A.1.4).


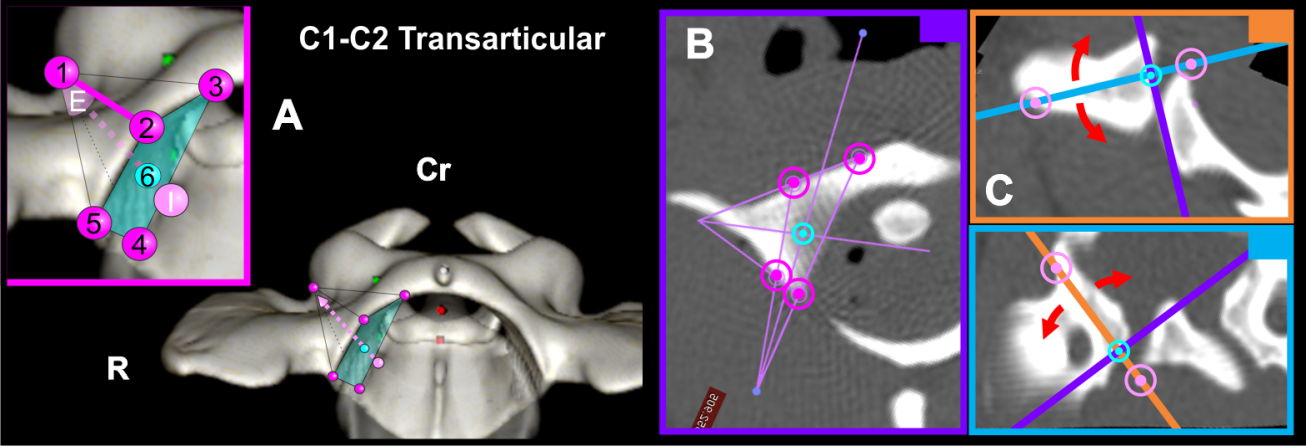


1. Step-by-step method of determination of C1-C2 transarticular optimal implant.
   (A) 3D reconstruction showing C1 pedicle simplified into a pyramid, (B) Geometrical determination of the center of the pyramid base, (C) Optimal axis determined by centering its position dorsoventrally and lateromedially rotating around the center.
   Point 1 is first positioned in 3D-VR at the medial portion of the alar notch. Point 2 is then positioned in 3D-VR mode by tracing a line passing through 1 and the medial border of the craniomedial border of the alar foramen. Point 2 is located at the intersection between this line and the ventral ridge of the caudal articular surface of C1. Point 3 is located in 3D-VR at the ventromedial extremity of the caudal articular surface of C1. Point 4 is placed in 3D-VR at the caudodorsal extremity of the same articular surface. Point 5 is placed using 3D-MPR in the plane 2,3,4 to delineate the laterodorsal extremity of a pyramid. The center of the base 6 is then geometrically determined in 3D-MPR and the optimal implant placement is localized by rotating the axis around this center both dorsoventrally and lateromedially. Note that the positioning of the insertion point on C2 is somewhat imprecise due to slight misapposition between C1 and C2.


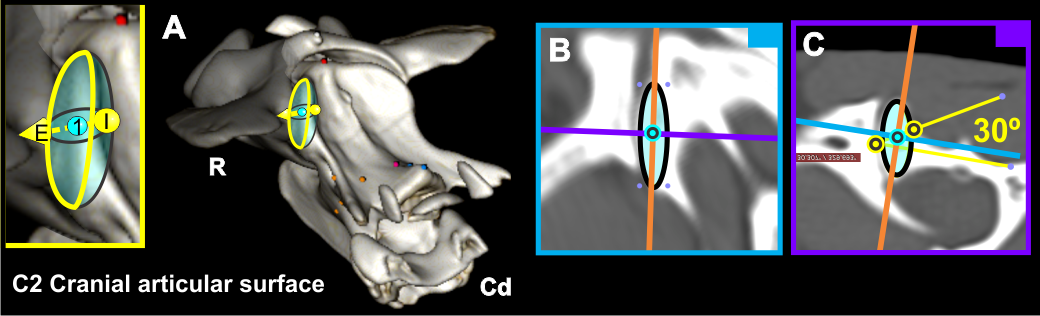


1. Step-by-step method of determination of C2 cranial articular surface optimal implant.
   (A) 3D reconstruction showing C2 cranial articular surface simplified into a semi-ellipsoid (in yellow), (B and C) Geometrical determination of the center (1) of the semi-ellipsoid using 2 ellipses placed in 2 orthogonal planes (black ellipses), (C) Optimal axis placed at 30° from a dorsal plane and passing through the center of the simplified corridor.

Determination of C2 pedicular optimal implantation axis

The method of geometrical simplification for C2 pedicles is similar to C1 pedicular OSIC. The main difference is that the vertebral artery cannot be avoided at this level due to extremely thin pedicles. As a result, the transverse foramen was included into the OSIC and optimal implants were knowingly passing through the foramen (Fig A.1.5).

Determination of parasagittal C2 caudal vertebral body optimal implantation axis

The caudal vertebral body of C2 has a pyramidal shape naturally. However, the base located at the level of the epiphysis is concave due to intervertebral disc articulation. This slight irregularity was considered in the process of 3D simplification by defining the sagittal plane as a bisector plane of the corridor as depicted in Fig A.1.6.


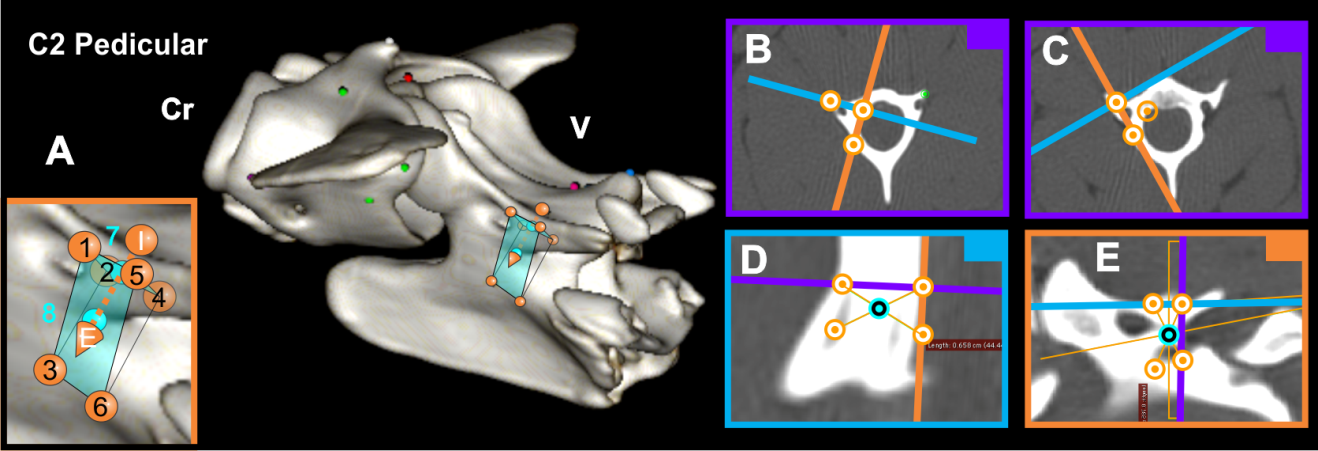


1. Step-by-step method of determination of C2 pedicular optimal implant.
   (A) 3D reconstruction showing C2 pedicle simplified into prism, (B and C) Positioning of ROI landmarks are initially performed in the transverse plane, (D and E) Geometrical determination of the ventral and lateral surfaces of the prism similar to C1 pedicle. All points are placed using 3D-MPR for this OSIC. Point 1,2 and 3 are first positioned in the transverse plane at the level of the cranial extremity of the transverse foramen. 1 is defined as the lateroventral margin of the pedicle, 2 is defined as the lateroventral margin of the vertebral foramen and 3 is defined as the dorsal margin of the pedicle identified by drawing a line tangent to the vertebral foramen passing through 2. Point 4 is placed similarly to 2 but at the level of the caudal extremity of the transverse foramen. Then, the plane 1,2,4 is identified in 3D-MPR and 5 is positioned to identify the laterocaudal limit of the ventral surface of the prism. Similarly, the plane 1,3,5 is localized and 6 is placed caudodorsally. The optimal implant is defined as the line passing through both ventral and lateral surface centers 7 and 8.


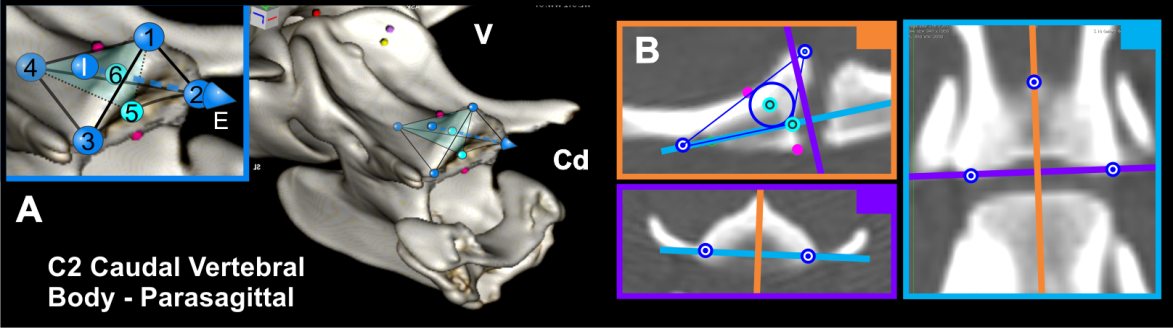


1. Step-by-step method of determination of C2 caudal vertebral body (parasagittal) optimal implant.
   (A) 3D reconstruction showing C2 caudal vertebral body simplified into a pyramid with concave basis, (B) Geometrical determination of the center of the simplified corridor in sagittal plane. Point 1, 2 and 3 were placed in 3D-VR with 1 being the ventral eminence, and 2-3 the lateral extremities of C2 epiphysis. Point 4 was placed using 3D-MPR in the sagittal plane at the intersection between the ventral limit of the vertebral foramen and a line tangent to the ventral surface of C2 passing through 1. The point 5 was defined in 3D-MPR as the intersection between the 2,3,4 plane and the caudal extremity of C2 epiphysis sagitally. The center of the triangle 1,4,5 was then geometrically determined and the optimal axes defined as the line 6-2 for the right implant and 6-3 for the left.


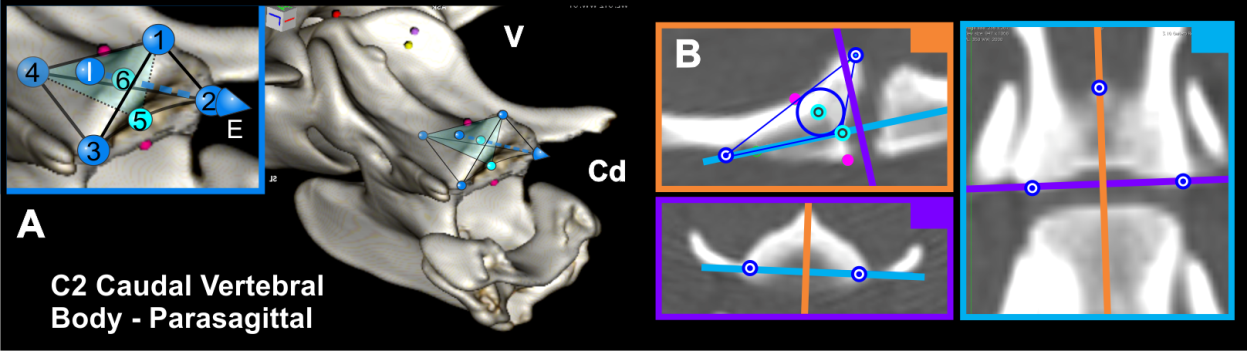


Determination of sagittal C1 and C2 optimal implantation axis

The determination of optimal implant positions located in the sagittal plane did not require 3D geometrical simplification as the optimal position was predefined within 2 dimensions. Instead an insertion point was subjectively defined for C1 ventral arch and C2 cranial vertebral body implants, while an exit point was predefined for C2 caudal vertebral body implants. This allowed positioning the optimal implant in space by determining the sagittal bisector of each corridor (Fig A.1.7).


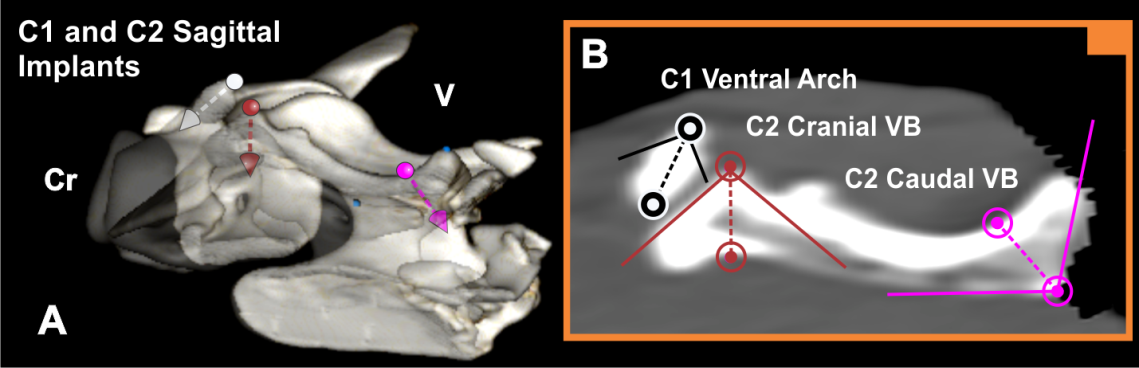


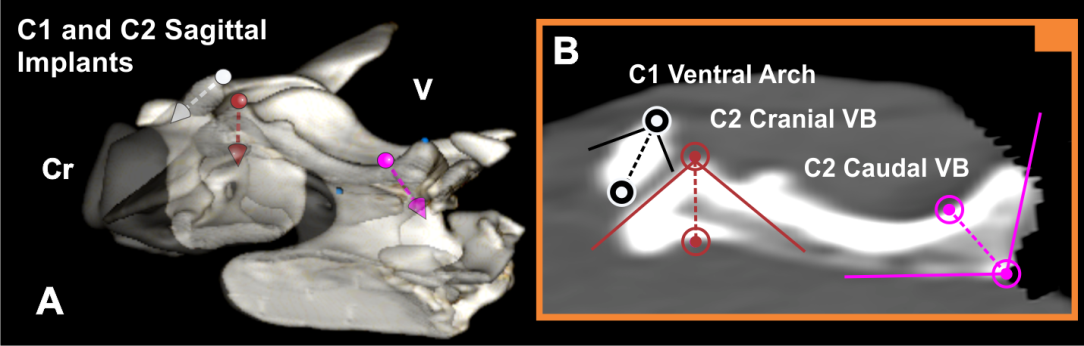


1. Step-by-step method of determination of C1 and C2 sagittal optimal implants.
   (A) 3D reconstruction showing the typical location of the 3 sagittal implants, (B) Geometrical determination of each optimal axis using the bisector method. For C1 ventral arch and C2 cranial vertebral body the angular width of the bone corridor was measured from the insertion point perspective, while the exit point was used for C2 caudal vertebral body. The bisector lines of these angles define the optimal implant positions.
