# Supplementary material for: Canine atlantoaxial optimal safe implantation corridors – description and validation of a novel 3D presurgical planning method using OsiriX™
Source: BMC Vet Res. 2016 Sep 6;12(1):188. doi: 10.1186/s12917-016-0824-3 (PMC5012052; doi:10.1186/s12917-016-0824-3)
Supplement: Additional file 2: — Cartesian vectorial calculations used to determine projected angles of optimal implants. Step by step demonstration of the mathematical equations used for semi-automation of the method. (DOCX 4598 kb) [file 12917_2016_824_MOESM2_ESM.docx]

ADDITIONAL FILE 2:
CARTESIAN VECTORIAL CALCULATIONS USED TO DETERMINE
PROJECTED ANGLES OF OPTIMAL IMPLANTS

Notations

$\mathbb{R}^{3}$: Three-dimensional real coordinate space

. : Multiplication

× : Cross product of vectors

: Dot product of vectors
$\vec{X}$: vector X
$\left\| X \right\|$: norm of vector X
$\hat{X}$: unit vector X (normalized)
${( )}_{X} or {[ ]}_{X}$ : coordinates of a point or vector defined with respect to a basis X
$\vartheta_{Anatomical plane(\hat{X})}$ : projected angle on an anatomical plane with respect to the vector $\hat{X}$

Definition of anatomical coordinate systems

A Cartesian 3D coordinate system is most commonly used to define spatial relationships in$\mathbb{R}^{3}$. A CT study provides 3D representation of a finite space which is the sum of all the slices of the anatomical region studied. The basis (or coordinate system) for the CT space is defined by 3 orthogonal unit vectors *(*$\hat{i}, \hat{j}, \hat{k}$*)* and an origin${O \left( 0, 0, 0 \right)}_{CT}$. By definition, the coordinates of the basis unit vectors are$\hat{i} \left( 1, 0, 0 \right)_{CT}, \hat{j} \left( 0, 1, 0 \right)_{CT},$ and $\hat{k} \left( 0, 0, 1 \right)_{CT}.$The basis $(\hat{i}, \hat{j}, \hat{k})$is then used to define any vector $\vec{u} \left( x, y, z \right)_{CT}$ within the CT space as a linear combination of the basis vectors. This is represented by the equation$\vec{u}=x . \hat{i}+y . \hat{j}+z . \hat{k}$; which is basically the definition of coordinates relative to a basis. In our study, the coordinates from OsiriX™ are provided with respect to the CT basis which has little anatomical meaning. These coordinates can be used to calculate non-oriented dimensional relationships (distances or absolute angles), but equations to define spatial relationships with respect to anatomical axes and planes (projected distances and angles) would be extremely complicated if used as is.

In order to define spatial relationships relative to anatomical structures (in our case the vertebrae C1 and C2), a change of coordinate systems is applied which greatly simplify the equations. An anatomical Cartesian coordinate system can be defined by 3 unit vectors$\left( \hat{i_{1}}, \hat{j_{1}}, \hat{k_{1}} \right)$and a subjective origin ${O_{1} (0, 0, 0)}_{C1}$ for C1 and similarly $\left( \hat{i_{2}}, \hat{j_{2}}, \hat{k_{2}} \right)$and $O_{2}$ ${(0, 0, 0)}_{C2}$ for C2. These vectors are chosen so that $\hat{i_{1}} / \hat{i_{2}}$are direction vectors of C1 and C2 craniocaudal axes, $\hat{j_{1}} / \hat{j_{2}}$are direction vectors of the ventrodorsal axes and $\hat{k_{1}} / \hat{k_{2}}$ are direction vectors of the right to left axes. This will define 2 “right-handed” coordinate systems respectively for C1 and C2. In practice, this is achieved by selecting 3 points within the sagittal plane of the vertebra of interest.

For C1, the origin of the system $(O_{1})$ is placed at the level of the ventral tuberosity, whereas the other 2 points $A_{1}$ and $B_{1}$are placed along the craniocaudal axis. The ventrodorsal axis of C1 is defined as the line passing through the cranial margin of the dorsal and ventral arch in the sagittal plane. The craniocaudal axis is defined as its perpendicular in the same plane. For C2, the origin $(O_{2})$ is placed in the sagittal plane, at the cranioventral eminence located at the base of the dens. The other 2 points $A_{2}$ and $B_{2}$ are placed along the craniocaudal axis defined as the sagittal ventral border of the vertebral foramen. The following equations will demonstrate how the change of coordinate system is operated on the CT system values in order to calculate projected angles relative to the anatomical planes of the vertebrae (sagittal, transverse and dorsal). C1 is used as an example, but the equations are identical for C2.

Determination of the coordinates of the C1 basis vectors in the CT system

To simplify the equations, the unit vectors' coordinates with respect to the CT base will be noted as follows:

$${\left[ \hat{i_{1}} \right]_{CT}= \left[ \begin{matrix} X_{\hat{i_{1}}} \\ Y_{\hat{i_{1}}} \\ Z_{\hat{i_{1}}} \end{matrix} \right]_{CT} , \left[ \hat{j_{1}} \right]}_{CT}= \left[ \begin{matrix} X_{\hat{j_{1}}} \\ Y_{\hat{j_{1}}} \\ Z_{\hat{j_{1}}} \end{matrix} \right]_{CT} and \left[ \hat{k_{1}} \right]_{CT}= \left[ \begin{matrix} X_{\hat{k_{1}}} \\ Y_{\hat{k_{1}}} \\ Z_{\hat{k_{1}}} \end{matrix} \right]_{CT}$$

By definition, $\vec{A_{1}B_{1}}$has the same direction as $\hat{i_{1}}$and therefore $\left[ \hat{i_{1}} \right]_{CT}$can be directly calculated from the following equation:

$$\left[ \hat{i_{1}} \right]_{CT}=\left[ \hat{A_{1}B_{1}} \right]_{CT}=\frac{\left[ \vec{A_{1}B_{1}} \right]_{CT}}{\left\| A_{1}B_{1} \right\|}$$

$$=\frac{1}{\sqrt{\left( x_{B1}{- x}_{A1} \right)^{2}+\left( y_{B1}{- y}_{A1} \right)^{2}+ \left( z_{B1}{- z}_{A1} \right)^{2}}} . \left[ \begin{matrix} x_{B1}{- x}_{A1} \\ y_{B1}{- y}_{A1} \\ z_{B1}{- z}_{A1} \end{matrix} \right]_{CT}$$

In other words, $X_{\hat{i_{1}}}, Y_{\hat{i_{1}}}$ and $Z_{\hat{i_{1}}}$can be directly calculated from $A_{1}$ and $B_{1}$ coordinates given by OsiriX.

$\hat{k_{1}}$is the normal vector of the sagittal plane (i.e. right to left axis is perpendicular to the sagittal plane). The cross product $\hat{i_{1}} \times\vec{O_{1}A_{1}}$ can therefore be used to determine $\left[ \hat{k_{1}} \right]_{CT}$.

$$\left[ \hat{k_{1}} \right]_{CT}=\left[ \hat{\hat{i_{1}} \times\vec{O_{1}A_{1}}} \right]_{CT}=\frac{\left[ \hat{i_{1}} \times\vec{O_{1}A_{1}} \right]_{CT}}{\left\| \hat{i_{1}} \times\vec{O_{1}A_{1}} \right\|}$$

$$with \left[ \hat{i_{1}} \times\vec{O_{1}A_{1}} \right]_{CT}= \left[ \begin{matrix} Y_{\hat{i_{1}}} \left( z_{A1}{- z}_{O1} \right) - Z_{\hat{i_{1}}}\left( y_{A1}{- y}_{O1} \right) \\ Z_{\hat{i_{1}}} \left( x_{A1}{- x}_{O1} \right) - X_{\hat{i_{1}}}\left( z_{A1}{- z}_{O1} \right) \\ X_{\hat{i_{1}}} \left( y_{A1}{- y}_{O1} \right) - Y_{\hat{i_{1}}}\left( x_{A1}{- x}_{O1} \right) \end{matrix} \right]_{CT}$$

$$and \left\| \hat{i_{1}} \times\vec{O_{1}A_{1}} \right\|=\sqrt{\begin{aligned} \left( Y_{\hat{i_{1}}} \left( z_{A1}{- z}_{O1} \right)-Z_{\hat{i_{1}}}\left( y_{A1}{- y}_{O1} \right) \right)^{2} \\ +\left( Z_{\hat{i_{1}}} \left( x_{A1}{- x}_{O1} \right)-X_{\hat{i_{1}}}\left( z_{A1}{- z}_{O1} \right) \right)^{2} \\ +\left( X_{\hat{i_{1}}} \left( y_{A1}{- y}_{O1} \right)-Y_{\hat{i_{1}}}\left( x_{A1}{- x}_{O1} \right) \right)^{2} \end{aligned}}$$

In other words,$X_{\hat{k_{1}}}, Y_{\hat{k_{1}}}, Z_{\hat{k_{1}}}$can be directly calculated from $A_{1}, B_{1} and O_{1}$ coordinates given by OsiriX.

$\hat{j_{1}}$is the normal vector of the dorsal plane (i.e. ventrodorsal axis is perpendicular to the dorsal plane). The cross product $\hat{k_{1}} \times\hat{i_{1}}$ can therefore be used to determine $\left[ \hat{j_{1}} \right]_{CT}$.

$$\left[ \hat{j_{1}} \right]_{CT}=\left[ \hat{\hat{k_{1}} \times\hat{i_{1}}} \right]_{CT}=\frac{\left[ \hat{k_{1}} \times\hat{i_{1}} \right]_{CT}}{\left\| \hat{k_{1}} \times\hat{i_{1}} \right\|}$$

$$with \left[ \hat{k_{1}} \times\hat{i_{1}} \right]_{CT}= \left[ \begin{matrix} Y_{\hat{k_{1}}} Z_{\hat{i_{1}}} - Z_{\hat{k_{1}}}Y_{\hat{i_{1}}} \\ Z_{\hat{k_{1}}} X_{\hat{i_{1}}} - X_{\hat{k_{1}}}Z_{\hat{i_{1}}} \\ X_{\hat{k_{1}}} Y_{\hat{i_{1}}} - Y_{\hat{k_{1}}}X_{\hat{i_{1}}} \end{matrix} \right]_{CT}$$

$$and \left\| \hat{k_{1}} \times\hat{i_{1}} \right\|= \sqrt{\begin{aligned} \left( Y_{\hat{k_{1}}} Z_{\hat{i_{1}}} - Z_{\hat{k_{1}}}Y_{\hat{i_{1}}} \right)^{2} \\ +\left( Z_{\hat{k_{1}}} X_{\hat{i_{1}}} - X_{\hat{k_{1}}}Z_{\hat{i_{1}}} \right)^{2} \\ + \left( X_{\hat{k_{1}}} Y_{\hat{i_{1}}} - Y_{\hat{k_{1}}}X_{\hat{i_{1}}} \right)^{2} \end{aligned}}$$

In other words $X_{\hat{j_{1}}}, Y_{\hat{j_{1}}} and Z_{\hat{j_{1}}}$can be directly calculated from $\left[ \hat{i_{1}} \right]_{CT} and \left[ \hat{k_{1}} \right]_{CT}$

This completes the first step of the mathematical method which aimed to determine the coordinates of the C1 basis vectors $\left[ \hat{i_{1}} \right]_{CT}, \left[ \hat{j_{1}} \right]_{CT} and \left[ \hat{k_{1}} \right]_{CT}.$

Determination of the transformation matrices between the CT coordinate system and anatomical coordinate systems

The purpose of applying a change of coordinate system is to be able to define a vector with respect to a different basis. In our study the vectors of interest $\vec{IE}$ are representing implant positions defined by an insertion point (I) and an exit point (E), within OsiriX™.

$$\left[ \vec{IE} \right]_{CT}= \left[ \begin{matrix} X_{\vec{IE}} \\ Y_{\vec{IE}} \\ Z_{\vec{IE}} \end{matrix} \right]_{CT}; \left[ \vec{IE} \right]_{C1}= \left[ \begin{matrix} {X^{'}}_{\vec{IE}} \\ {Y^{'}}_{\vec{IE}} \\ {Z^{'}}_{\vec{IE}} \end{matrix} \right]_{C1}; \left[ \vec{IE} \right]_{C2}= \left[ \begin{matrix} {X^{''}}_{\vec{IE}} \\ {Y^{''}}_{\vec{IE}} \\ {Z^{''}}_{\vec{IE}} \end{matrix} \right]_{C2}$$

The direction cosine $\left[ DC \right]$ and direction cosine inverse $\left[ DC \right]^{-1}$ of a particular anatomical coordinate system are defined by the following equations:

$$\left[ \vec{IE} \right]_{CT}= \left[ \begin{matrix} X_{\vec{IE}} \\ Y_{\vec{IE}} \\ Z_{\vec{IE}} \end{matrix} \right]_{CT}=\left[ {DC}_{C1} \right] . \left[ \begin{matrix} {X^{'}}_{\vec{IE}} \\ {Y^{'}}_{\vec{IE}} \\ {Z^{'}}_{\vec{IE}} \end{matrix} \right]_{C1}= {\left[ {DC}_{C2} \right] . \left[ \begin{matrix} {X^{''}}_{\vec{IE}} \\ {Y^{''}}_{\vec{IE}} \\ {Z^{''}}_{\vec{IE}} \end{matrix} \right]}_{C2}$$

$$\left[ \vec{IE} \right]_{C1}=\left[ \begin{matrix} {X^{'}}_{\vec{IE}} \\ {Y^{'}}_{\vec{IE}} \\ {Z^{'}}_{\vec{IE}} \end{matrix} \right]_{C1}= \left[ {DC}_{C1} \right]^{-1} . \left[ \begin{matrix} X_{\vec{IE}} \\ Y_{\vec{IE}} \\ Z_{\vec{IE}} \end{matrix} \right]_{CT}$$

$$\left[ \vec{IE} \right]_{C2}=\left[ \begin{matrix} {X^{''}}_{\vec{IE}} \\ {Y^{''}}_{\vec{IE}} \\ {Z^{''}}_{\vec{IE}} \end{matrix} \right]_{C2}= \left[ {DC}_{C2} \right]^{-1} . \left[ \begin{matrix} X_{\vec{IE}} \\ Y_{\vec{IE}} \\ Z_{\vec{IE}} \end{matrix} \right]_{CT}$$

$$By definition \left[ {DC}_{C1} \right]^{-1}= \left[ \begin{matrix} \hat{i} \hat{i_{1}} & \hat{j} \hat{i_{1}} & \hat{k} \hat{i_{1}} \\ \hat{i} \hat{j_{1}} & \hat{j} \hat{j_{1}} & \hat{k} \hat{j_{1}} \\ \hat{i} \hat{k_{1}} & \hat{j} \hat{k_{1}} & \hat{k} \hat{k_{1}} \end{matrix} \right]$$

and$\left[ {DC}_{C1} \right]= \left[ \begin{matrix} \hat{i} \hat{i_{1}} & \hat{i} \hat{j_{1}} & \hat{i} \hat{k_{1}} \\ \hat{j} \hat{i_{1}} & \hat{j} \hat{j_{1}} & \hat{j} \hat{k_{1}} \\ \hat{k} \hat{i_{1}} & \hat{k} \hat{j_{1}} & \hat{k} \hat{k_{1}} \end{matrix} \right]$

$${Knowing that \left[ \hat{i} \right]}_{CT}= \left[ \begin{matrix} 1 \\ 0 \\ 0 \end{matrix} \right]_{CT}; \left[ \hat{j} \right]_{CT}= \left[ \begin{matrix} 0 \\ 1 \\ 0 \end{matrix} \right]_{CT}; \left[ \hat{k} \right]_{CT}= \left[ \begin{matrix} 0 \\ 0 \\ 1 \end{matrix} \right]_{CT}$$

and that$\vec{a} \vec{b}= x_{a} . x_{b}+ y_{a} . y_{b}+ z_{a} . z_{b}=\cos\vartheta$

$$(\vartheta being the angle between \vec{a} and \vec{b)}$$

This implies that $\left[ {DC}_{C1} \right]^{-1}= \left[ \begin{matrix} X_{\hat{i_{1}}} & Y_{\hat{i_{1}}} & Z_{\hat{i_{1}}} \\ X_{\hat{j_{1}}} & Y_{\hat{j_{1}}} & Z_{\hat{j_{1}}} \\ X_{\hat{k_{1}}} & Y_{\hat{k_{1}}} & Z_{\hat{k_{1}}} \end{matrix} \right]$

$$and that \left[ {DC}_{C1} \right]= \left[ \begin{matrix} \begin{matrix} X_{\hat{i_{1}}} \\ Y_{\hat{i_{1}}} \\ Z_{\hat{i_{1}}} \end{matrix} & \begin{matrix} X_{\hat{j_{1}}} \\ Y_{\hat{j_{1}}} \\ Z_{\hat{j_{1}}} \end{matrix} & \begin{matrix} X_{\hat{k_{1}}} \\ Y_{\hat{k_{1}}} \\ Z_{\hat{k_{1}}} \end{matrix} \end{matrix} \right]$$

Therefore we can determine $\left[ {DC}_{C1} \right]^{-1}$ and $\left[ {DC}_{C1} \right]$ by knowing$\left[ \hat{i_{1}} \right]_{CT}, \left[ \hat{j_{1}} \right]_{CT} and \left[ \hat{k_{1}} \right]_{CT}$ which completes the second step of the mathematical method.

Determination of implant vector coordinates with respect to the anatomical basis $\boldsymbol{(i. e.}\left[ \vec{\boldsymbol{IE}} \right]_{\boldsymbol{C}\boldsymbol{1}}$ or $\left[ \vec{\boldsymbol{IE}} \right]_{\boldsymbol{C}\boldsymbol{2}}\boldsymbol{)}$

$$\left[ \vec{IE} \right]_{C1}=\left[ \begin{matrix} {X^{'}}_{\vec{IE}} \\ {Y^{'}}_{\vec{IE}} \\ {Z^{'}}_{\vec{IE}} \end{matrix} \right]_{C1}=\left[ {DC}_{C1} \right]^{-1} . \left[ \begin{matrix} X_{\vec{IE}} \\ Y_{\vec{IE}} \\ Z_{\vec{IE}} \end{matrix} \right]_{CT}$$

$$\left[ \begin{matrix} {X^{'}}_{\vec{IE}} \\ {Y^{'}}_{\vec{IE}} \\ {Z^{'}}_{\vec{IE}} \end{matrix} \right]_{C1}=\left[ \begin{matrix} X_{\hat{i_{1}}} & Y_{\hat{i_{1}}} & Z_{\hat{i_{1}}} \\ X_{\hat{j_{1}}} & Y_{\hat{j_{1}}} & Z_{\hat{j_{1}}} \\ X_{\hat{k_{1}}} & Y_{\hat{k_{1}}} & Z_{\hat{k_{1}}} \end{matrix} \right] . \left[ \begin{matrix} x_{E}- x_{I} \\ y_{E}- y_{I} \\ z_{E}- z_{I} \end{matrix} \right]_{CT}$$

$$\left[ \begin{matrix} {X^{'}}_{\vec{IE}} \\ {Y^{'}}_{\vec{IE}} \\ {Z^{'}}_{\vec{IE}} \end{matrix} \right]_{C1}=\left[ \begin{matrix} X_{\hat{i_{1}}} . \left( x_{E}- x_{I} \right)+Y_{\hat{i_{1}}}{. (y}_{E}- y_{I})+ Z_{\hat{i_{1}}} . (z_{E}- z_{I}) \\ X_{\hat{j_{1}}} . \left( x_{E}- x_{I} \right)+{Y_{\hat{j_{1}}} . (y}_{E}- y_{I})+ Z_{\hat{j_{1}}} . (z_{E}- z_{I}) \\ X_{\hat{k_{1}}} . \left( x_{E}- x_{I} \right)+Y_{\hat{k_{1}}}{. (y}_{E}- y_{I})+ Z_{\hat{k_{1}}} . (z_{E}- z_{I}) \end{matrix} \right]_{C1}$$

Therefore we can determine $\left[ \vec{IE} \right]_{C1}$ by knowing$\left[ \hat{i_{1}} \right]_{CT}, \left[ \hat{j_{1}} \right]_{CT}, \left[ \hat{k_{1}} \right]_{CT}$ and $\left[ \vec{IE} \right]_{CT}$ which completes the third step of our mathematical method. Similar equations are valid for $\left[ \vec{IE} \right]_{C2}$

Determination of implant projected angles with respect to the anatomical planes

Projected angles are directly calculated from $\left[ \vec{IE} \right]_{C1}$ and $\left[ \vec{IE} \right]_{C2}$ using the following formulas:

$\vartheta_{Sagittal (\hat{i_{1}})}=\tan^{-1} \left( \frac{{Y^{'}}_{\vec{IE}}}{{X^{'}}_{\vec{IE}}} \right); \vartheta_{Sagittal (\hat{j_{1}})}=\tan^{-1} \left( \frac{{X^{'}}_{\vec{IE}}}{{Y^{'}}_{\vec{IE}}} \right)$

$\vartheta_{Transverse (\hat{j_{1}})}=\tan^{-1} \left( \frac{{Z^{'}}_{\vec{IE}}}{{Y^{'}}_{\vec{IE}}} \right); \vartheta_{Transverse (\hat{k_{1}})}=\tan^{-1} \left( \frac{{Y^{'}}_{\vec{IE}}}{{Z^{'}}_{\vec{IE}}} \right)$

$\vartheta_{Dorsal (\hat{i_{1}})}=\tan^{-1} \left( \frac{{Z^{'}}_{\vec{IE}}}{{X^{'}}_{\vec{IE}}} \right); \vartheta_{Dorsal (\hat{k_{1}})}=\tan^{-1} \left( \frac{{X^{'}}_{\vec{IE}}}{{Z^{'}}_{\vec{IE}}} \right)$
